# Supplementary figures and images for: Alleviating chronic ER stress by p38-Ire1-Xbp1 pathway and insulin-associated autophagy in C. elegans neurons
Source: PLoS Genet. 2020 Sep 28;16(9):e1008704. doi: 10.1371/journal.pgen.1008704 (PMC7544145; doi:10.1371/journal.pgen.1008704)

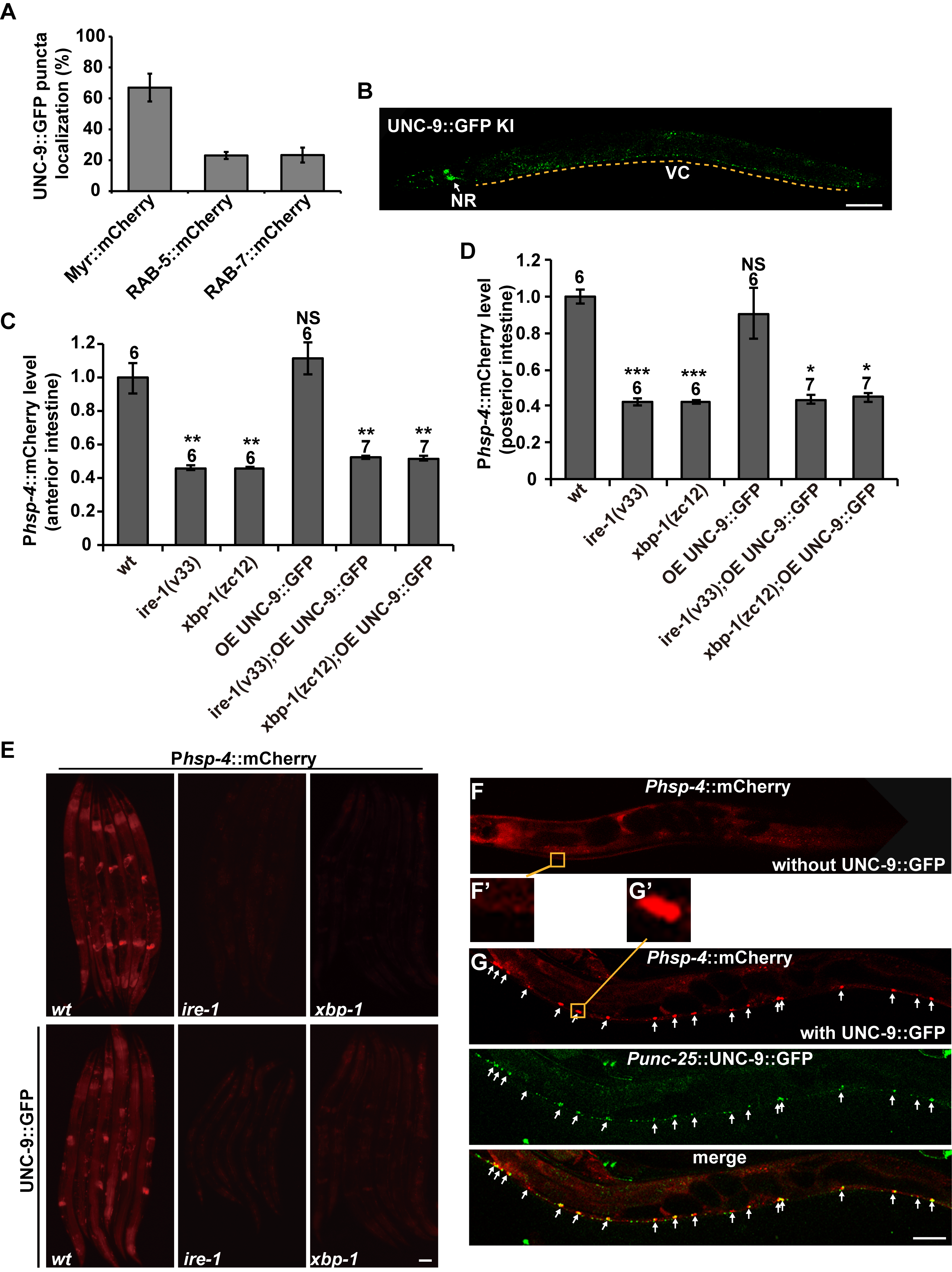

Supplement: S1 Fig — (A) Quantification of the UNC-9::GFP co-distribution with Myr::mCher, RAB-5:mChery or RAB-7::mCherry. N = 5. (B) A worm-scale image of UNC-9::GFP knock in (KI) (green). NR, nerve ring. VC, ventral cord. (C) Quantification of Phsp-4::mCherry signal in the anterior intestine region. (D) Quantification of Phsp-4::mCherry signal in the posterior intestine region. NS, not significant; **P < 0.01, *P < 0.05. One-way ANOVA with Dunnett’s test. (E) Expression of Phsp-4::mCherry (red) in wild-type (wt), ire-1 and xbp-1 animals with or without UNC-9::GFP. Scale bar represents 25 μm. (F) Phsp-4::mCherry distribution in wild type with no UNC-9::GFP overexpression in DD/VD cells. F’ is enlarged from F (boxed region). (F) Phsp-4::mCherry (red) is widely and weakly expressed in multiple tissues. F’ is enlarged from F (boxed region) showing a part of ventral cord region. (G) Phsp-4::mCherry (red) is induced in UNC-9::GFP (green) expressing DD/VD neurons (white arrows). G’ is enlarged from G (boxed region) showing a DD/VD cell (red). Scale bar represents 50 μm (TIF) [file pgen.1008704.s001.tif]

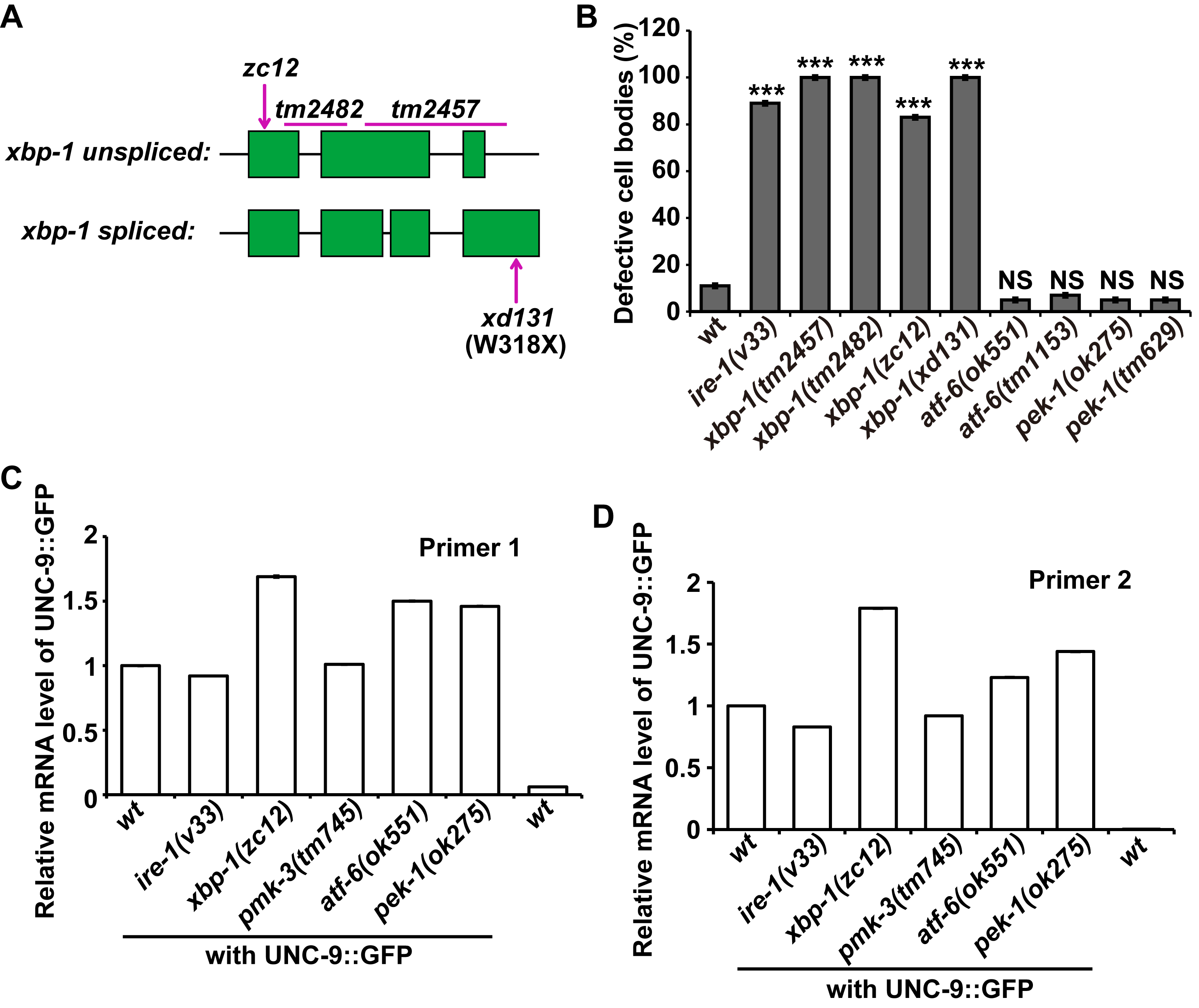

Supplement: S2 Fig — (A) The structure of the xbp-1 gene. The molecular lesions are indicated for the zc12, tm2482, tm2457, and xd131 alleles. Green boxes indicate exons. (B) Quantification of the UNC-9::GFP localization defect in various genotypes. N = 20 for each genotype; ***P < 0.001; **P < 0.01. One-way ANOVA with Dunnett’s test. (C and D) Quantification of the mRNA expression of Punc-25::UNC-9::GFP in various genotypes. Three independent biological repeats. (TIF) [file pgen.1008704.s002.tif]

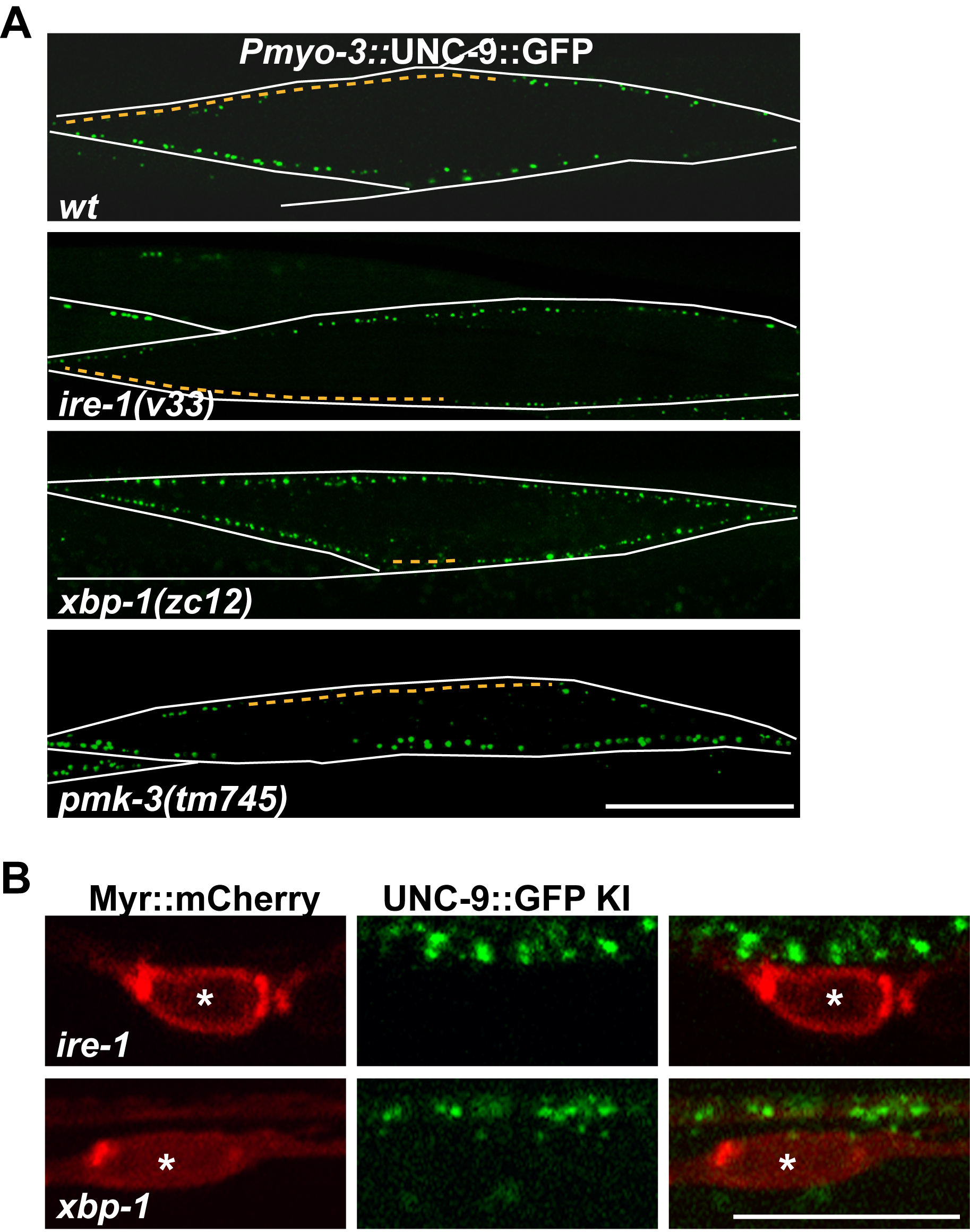

Supplement: S3 Fig — (A) The localization of UNC-9::GFP (green) in muscle cells in wild-type (wt), ire-1(v33), xbp-1(zc12), and pmk-3(tm745) animals carrying the Pmyo-3::UNC-9::GFP transgene. White lines highlight the muscle cells. Dashed lines highlight the regions from which the UNC-9::GFP puncta are absent. Scale bar represents 25 μm. (B) UNC-9::GFP expressed at the endogenous level from a knock-in allele (UNC-9::GFP KI) is distributed on the neuronal processes and cell surface of DD/VD neurons (asterisks) in ire-1 (A) and xbp-1 (B)mutants. Scale bar represents 5 μm. (TIF) [file pgen.1008704.s003.tif]

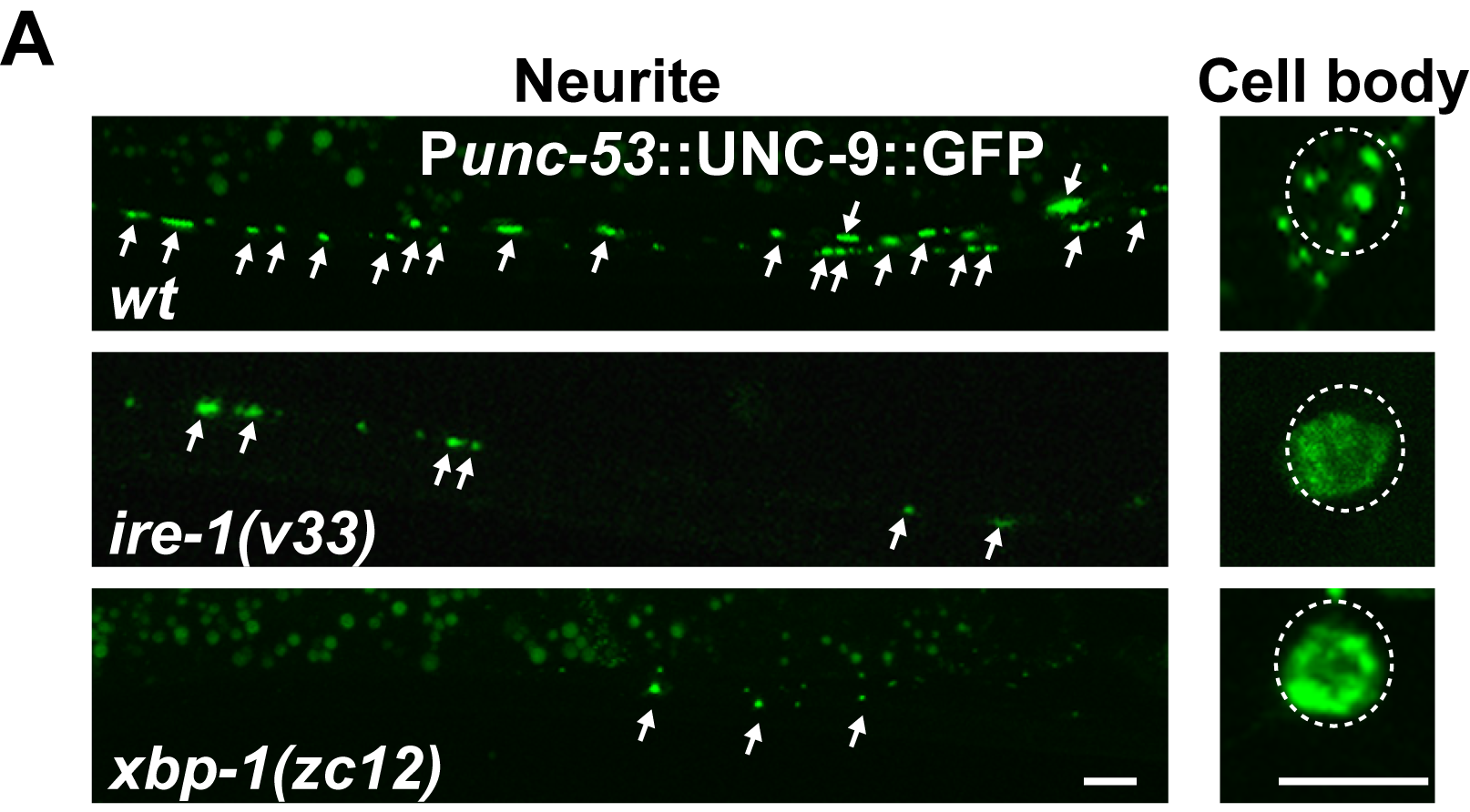

Supplement: S4 Fig — (A) The localization of UNC-9::GFP in Punc-53 expressing neurons (green) in wild-type (wt), ire-1(v33), and xbp-1(zc12) animals carrying the Punc-53::UNC-9::GFP transgene. White arrows indicate UNC-9::GFP puncta on neurites. Dashed lines encircle the cell bodies. Scale bars represent 5 μm. (TIF) [file pgen.1008704.s004.tif]

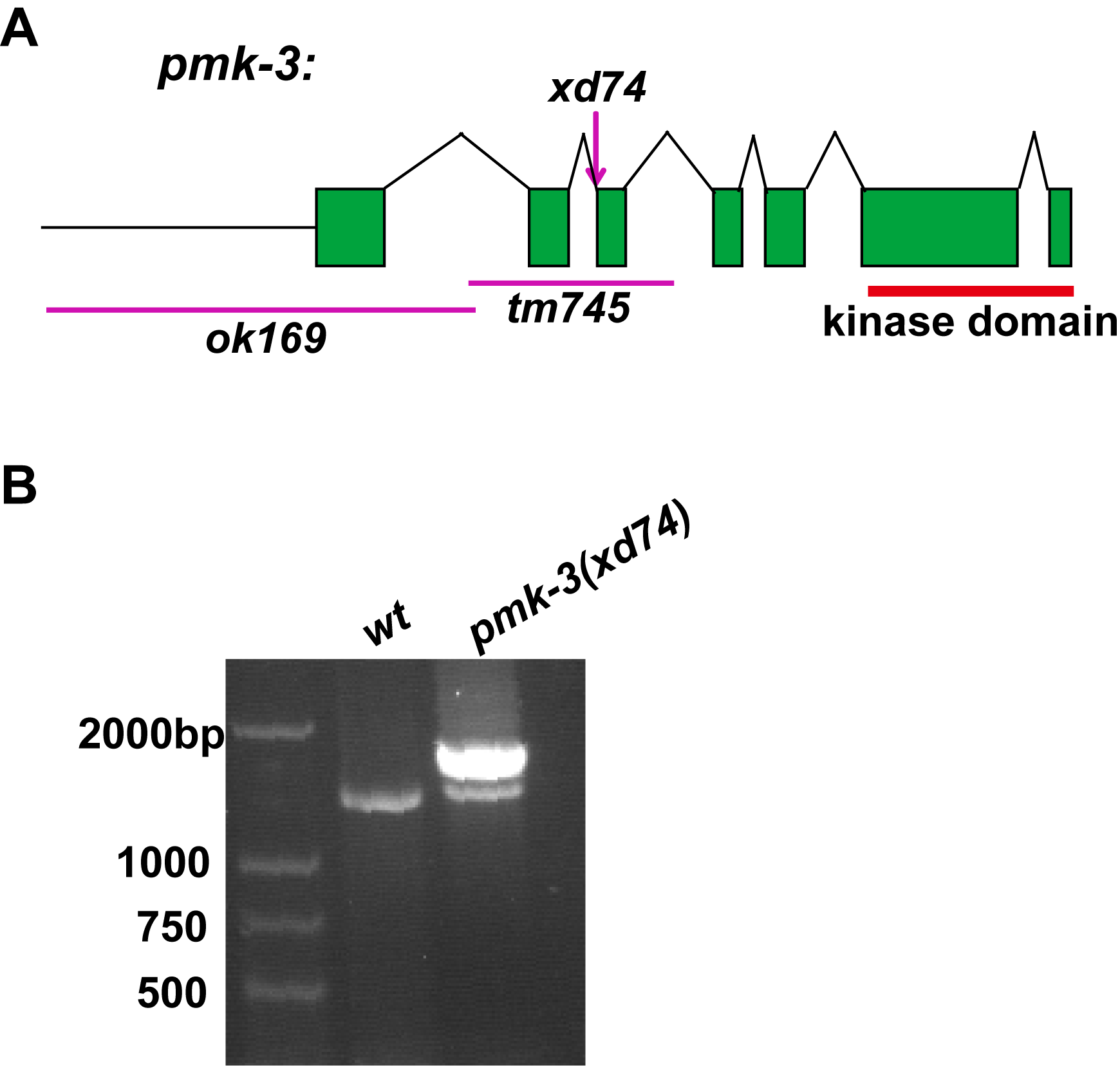

Supplement: S5 Fig — (A) The structure of the pmk-3 gene. The molecular lesions are indicated for the ok169, tm745 and xd74 alleles. The kinase domain is indicated. Green boxes indicate exons. (B) The RT-PCR results for pmk-3 in wild type and pmk-3(xd74). (TIF) [file pgen.1008704.s005.tif]

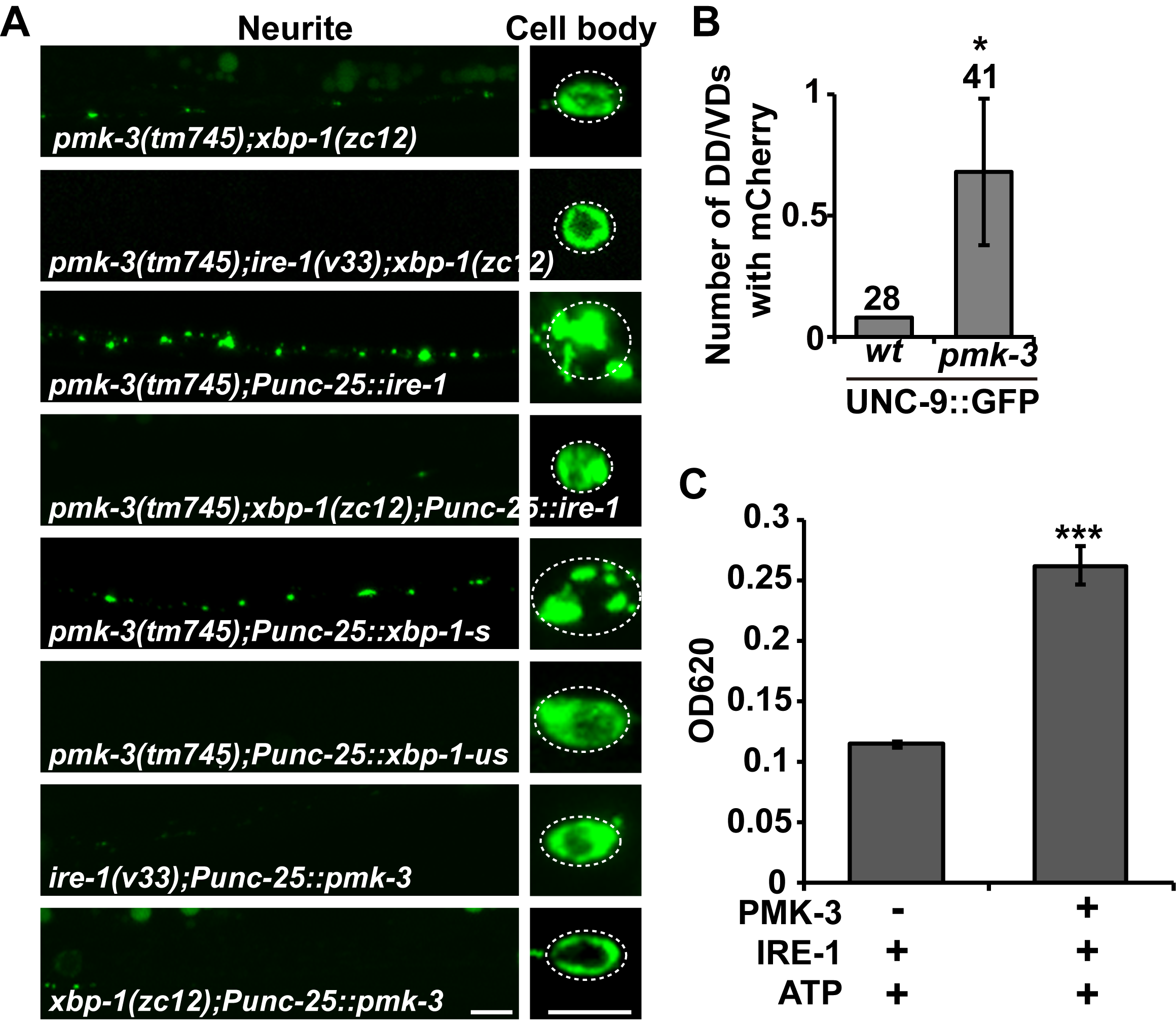

Supplement: S6 Fig — (A) The localization of UNC-10::GFP (green) in DD/VDs in various genotypes. Dashed lines encircle the cell bodies. Scale bars represent 5 μm. (B) Alternative splicing of xbp-1 represented by mCherry signal in wild-type (wt) and pmk-3 animals. N is indicated for each genotype. (C) The in vitro kinase assay. ***P < 0.001. Student’s t-test. Three independent biological repeats. (TIF) [file pgen.1008704.s006.tif]

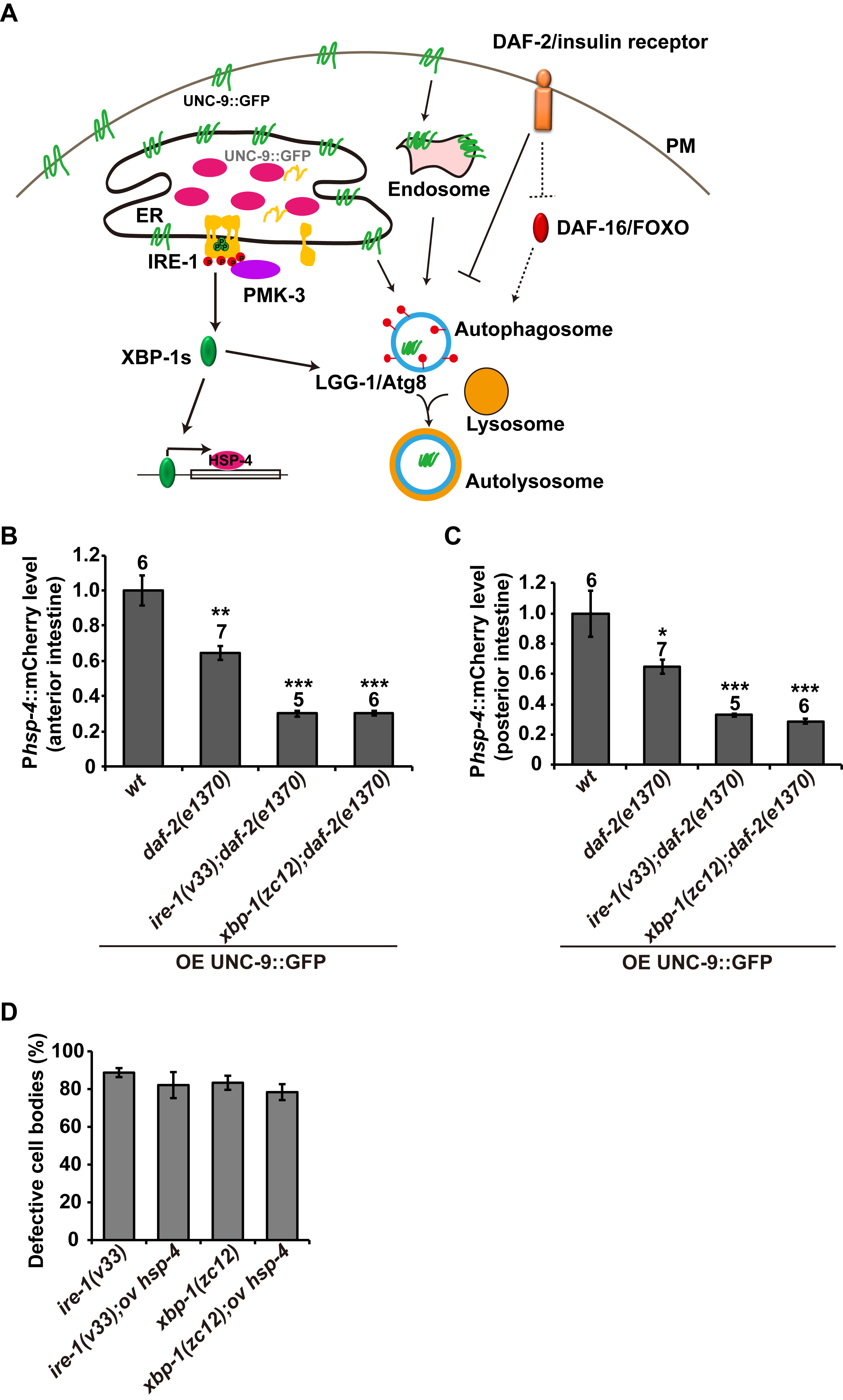

Supplement: S7 Fig — (A) The PMK-3-IRE-1-XBP-mediated UPR acts in parallel with insulin-inhibited autophagy to alleviate chronic stress induced by excess UNC-9::GFP proteins. (B) Quantification of Phsp-4::mCherry signal in the anterior intestine region. (C) Quantification of Phsp-4::mCherry signal in the posterior intestine region. ***P < 0.001; **P < 0.01; *P < 0.05; NS, not significant. One-way ANOVA with Dunnett’s test. (D) The overexpression of hsp-4 does not suppress the mutant phenotype of ire-1(v33) or xbp-1(zc12) animals. (TIF) [file pgen.1008704.s007.tif]
