# Supplementary material for: Alleviating chronic ER stress by p38-Ire1-Xbp1 pathway and insulin-associated autophagy in C. elegans neurons
Source: PLoS Genet. 2020 Sep 28;16(9):e1008704. doi: 10.1371/journal.pgen.1008704 (PMC7544145; doi:10.1371/journal.pgen.1008704)
Supplement: S1 Table — (DOCX) [file pgen.1008704.s008.docx]

**S1 Table: The roles of MAPK components in UNC-9::GFP localization.**

| Genes | Alleles used | UNC-9::GFP localization defect |
| --- | --- | --- |
| *pmk-1* | *km25* | - |
| *pmk-3* | *ok169* | + |
|  | *tm745* | + |
|  | *xd74* | + |
| *jnk-1* | *gk7* | - |
| *kgb-1* | *um3* | - |
| *kgb-2* | *gk361* | - |
| *jkk-1* | *km2* | - |
| *mek-1* | *ks54* | - |
| *sek-1* | *km4* | - |
| *mkk-4* | *ok1545* | - |
| *sek-3* | *tm1344* | - |
| *sek-6* | *tm4305 tm4136* | - |
| *mtk-1* | *ok1382* | - |
| *nsy-1* | *ok593* | - |
| *mlk-1* | *ok2471* | - |
| *dlk-1* | *ju476* | - |
| *kin-18* | *ok395* | - |

“-” with no defect; “+” with defect
