# Supplementary material for: Alleviating chronic ER stress by p38-Ire1-Xbp1 pathway and insulin-associated autophagy in C. elegans neurons
Source: PLoS Genet. 2020 Sep 28;16(9):e1008704. doi: 10.1371/journal.pgen.1008704 (PMC7544145; doi:10.1371/journal.pgen.1008704)
Supplement: S2 Table — (DOCX) [file pgen.1008704.s009.docx]

**Supplementary Table 2. Transgenic strains**

| **Transgenes** | **DNA constructs** |
| --- | --- |
| XD1201  XD1317  XD3619  XD2486  XD4603  XD4617  XD4618  XD3900  XD3901  XD3949  XD3926  XD3729  XD3878  XD3879  XD3792  XD3274  XD3949  XD3115  XD3117  XD3783  XD3927  XD2533  XD3119  XD2533  XD3290  XD3792  XD3576  XD2829  XD2830  XD2840  XD2841  XD2842  XD2844  XD2891  XD2899  XD3257  XD1813  XD4006  XD3935  XD3413  XD2933  XD3361  XD3355  XD1681  XD2127  XD3421  XD3452  XD2675  XD2672  XD2673  XD2732  XD3348  XD3350  XD3352  XD3354  XD3356  XD3357  XD3389  XD3390  XD3391  XD3400  XD3401  XD3409  XD3410  XD3411  XD3412  XD3420  XD3472  XD3584  XD3398  XD3639  XD3795  XD3796  XD3929  XD3930  XD4004  XD4005  XD4007  XD4193  XD3794  XD4759  XD3639  XD3638  XD3794  XD4532  XD4533  XD4656 XD4658 XD4659  XD4618  XD4425  XD4522  XD4523  XD4525  XD4539  BCN1071  XD5038 XD5073 XD5074 XD5127 XD5087  XD4953  XD5085  XD5426  XD5545  XD5090 XD4946 XD4948 XD5159 XD5188 XD5246 XD5247 XD5248 XD5189 XD5190 XD5259  XD5293 XD5329  XD5330 XD5331 XD5332 XD5333 XD5125 XD5126 | *xdIs13:Punc-25::UNC-9::GFP*  *xdIs15:Punc-25::UNC-9::GFP*  *xdIs103: Pmyo-3::UNC-9::GFP*  *xdEx1116:Punc-25:UNC-10::GFP;Punc-25::SNB-1::mCherry*  *N2;xdEx2079(Punc-25::cytb-5.1::mCherry)*  *ire-1(v33);xdIs15;xdEx2082(Punc-25::cytb-5.1::mcherry)*  *xbp-1(zc12),xdIs15;xdEx2082(Punc-25::cytb-5.1::mcherry)*  *N2;xdEx1844(Punc-25::XBP-1us::mCherry)*  *xdIs15;xdEx1844(Punc-25::XBP-1us::mCherry)*  *ire-1(v33);xdIs15;xdEx1844(Punc-25::XBP-1us::mCherry)*  *pmk-3(tm745);xdIs15;* *xdEx1844(Punc-25::XBP-1us::mCherry)*  *ire-1(v33);xdIs15;xdEx1768 (Punc-25::XBP-1s)*  *ire-1(v33);xdIs15;xdEx1829(Punc-25::IRE-1)*  *ire-1(v33);xdIs15;xdEx1830(Pmyo-3::IRE-1)*  *ire-1(v33);xdIs15;xdEx1813(Punc-25::PMK-3 )*  *xbp-1(zc12);xdIs15;xdEx1564(Punc-25::XBP-1us)*  *xbp-1(zc12);xdIs15; xdEx1768(Punc-25::XBP-1s)*  *pmk-3(tm745);xdIs15;xdEx1455 (Punc-25::XBP-1us)*  *pmk-3(tm745);xdIs15;xdEx1457(Punc-25::XBP-1s)*  *pmk-3(tm745)xdIs15;xdEx1804(Punc-25::IRE-1)*  *pmk-3(tm745);xbp-1(zc12);xdIs15;xdEx1848(Punc-25::IRE-1)*  *pmk-3(tm745);xdIs15;xdEx1133(Punc-25::PMK-3)*  *pmk-3(tm745);xdIs15;xdEx1459(Punc-25::PMK-3TY)*  *pmk-3(xd74);xdIs15;xdEx1133(Punc-25::PMK-3)*  *xbp-1(zc12);xdIs15;xdEx1005(Punc-25::PMK-3)*  *ire-1(v33);xdIs15;xdEx1813(Punc-25::PMK-3)*  *xdEx1665(Punc-25::mCherry)*  *atf-6(ok551);xdIs15*  *pek-1(ok275);xdIs15*  *pek-1(tm629);xdIs15*  *xbp-1(tm2482);xdIs15*  *atf-6(tm1153);xdIs15*  *xbp-1(tm2457);xdIs15*  *xbp-1(xd131);xdIs15*  *ire-1(v33);xdIs13*  *xbp-1(zc12);xdIs15*  *xbp-1(xd131);xdIs15*  *ire-1(v33);xdIs103*  *xbp-1(zc12);xdIs103*  *pmk-3(tm745);xdIs103*  *xbp-1(xd131);juIs1*  *xbp-1(zc12);juIs1*  *xbp-1(zc12);hpIs61*  *pmk-3(xd74);xdIs15*  *pmk-3(tm745);xdIs15*  *pmk-3(ok169);xdIs15*  *pmk-3(tm745);juIs1*  *pmk-3(tm745);hpIs61*  *mek-1(ks54);xdIs15*  *sek-1(km4);xdIs15*  *mak-2(tm2927);xdIs15*  *nsy-1(ok593);xdIs13*  *kgb-2(gk361);xdIs15*  *mom-4(ne1539);xdIs15*  *dlk-1(tm4024);xdIs15*  *jkk-1(km2);xdIs15*  *mkk-4(ok1545);xdIs15*  *sek-1(km4);xdIs15*  *dlk-1(ju476);xdIs15*  *rpm-1(ju44ts);xdIs15*  *mek-1(ks54);xdIs15*  *mlk-1(ok2471);xdIs15*  *mtk-1(ok1382);xdIs15*  *dlk-1(tm4024);xdIs15*  *sek-6(tm4305 tm4136);xdIs15*  *jnk-1(gk7);xdIs15*  *sek-3(tm1344);xdIs15*  *pmk-1(km25);xdIs15*  *kgb-1(um3);xdIs15*  *xbp-1(zc12);pmk-3(tm745);xdIs15*  *pmk-3(tm745);xbp-1(zc12);xdIs15*  *ire-1(v33);pmk-3(tm745);xdIs15*  *pmk-3(tm745);ire-1(v33);xbp-1(zc12);xdIs15*  *xbp-1(xd131);daf-2(xd211);daf-16(mu86);xdIs15*  *pmk-3(tm745);daf-2(e1370);daf-16(mu86);xdIs15*  *ire-1(v33);daf-2(xd211);daf-16(mu86);xdIs15*  *ire-1(v33);daf-2(xd211);xdIs15*  *pmk-3(tm745);daf-2(xd211);xdIs15*  *pmk-3(tm745);daf-2(e1370);daf-16(mu86);xdIs15*  *ire-1(v33);daf-2(e1370);xdIs15*  *xbp-1(xd131);daf-2(e1370);xdIs15*  *xbp-1(zc12);daf-2(e1370);xdIs15*  *pmk-3(tm745);daf-2(e1370);xdIs15*  *ire-1(v33);daf-2(e1370);xdIs15*  *xdIs13;xdIs121:xdEx1665(Punc-25::mCherry)*  *ire-1(v33);xdIs15;xdEx1665(Punc-25::mCherry)*  *ire-1(v33);xdEx1665(Punc-25::mCherry)*  *xbp-1(zc12);xdIs15;xdEx1768(Punc-25::XBP-1s);Punc-25::XBP-1s;xdEx1665*  *ire-1(v33);xdIs15;xdEx1829(Punc-25::IRE-1);xdEx1665*  *ire-1(v33);xdIs15;xdEx1768(Punc-25::XBP-1s);xdEx1665*  *ire-1(v33);xdIs15;xdEx2005(Punc-25::IRE-1(T949A/S519G/S802G)*  *ire-1(v33);xdIs15;xdEx2032(Punc-25::IRE-1)*  *ire-1(v33);xdIs15;xdEx2033(Punc-25::IRE-1(S519G)*  *ire-1(v33);xdIs15;xdEx2035(Punc-25::IRE-1(T949A)*  *ire-1(v33);xdIs15;xdEx2042(Punc-25::IRE-1(S802G)*  *crgIs1006:Phsp-4::mCherry::unc-54 3'UTR*  *xdIs15;crgIs1006[Phsp-4::mCherry::unc-54 3'UTR]*  *xbp-1(zc12);xdIs15;crgIs1006[Pphsp-4::mCherry::unc-54 3'UTR]*  *pmk-3(tm745);xdIs15;crgIs1006[Phsp-4::mCherry::unc-54 3'UTR]*  *ire-1(v33);xdIs15;crgIs1006[Phsp-4::mCherry::unc-54 3'UTR]*  *xdIs15;xdEx2302(Punc-25::myr::mcherry)*  *xd430(UNC-9::GFP knock-in)* (outcrossed from PHX727 for 4 times)  *xd430(*UNC-9::GFP *knock-in);xdEx2300(Punc-25::myr::rmCherry)*  *ire-1(v33);* *xd430(UNC-9::GFP knock-in);xdEx2300(Punc-25::myr::rmCherry)*  *xbp-1(zc12);* *xd430(UNC-9::GFP knock-in);xdEx2300(Punc-25::myr::rmCherry)*  *xdIs15;xdEx2305(Punc-25::mCherry::MANS)*  *xdIs15;xdEx2224(Punc-25::mCherry::RAB-7)*  *xdIs15;xdEx2225(Punc-25::mCherry::RAB-5)*  *xdIs15;xdEx2318(Punc-25::mCherry::LGG-1)*  *N2;xdEx2318(Punc-25::mCherry::LGG-1)*  *ire-1(v33);xdIs15;xdEx2318(Punc-25::mCherry::LGG-1)*  *xbp-1(zc12);xdIs15;xdEx2318(Punc-25::mCherry::LGG-1)*  *pmk-3(tm745);xdIs15; xdEx2318(Punc-25::mCherry::LGG-1)*  *ire-1(v33);crgIs1006[Phsp-4::mCherry::unc-54 3'UTR]*  *xbp-1(zc12);crgIs1006[Phsp-4::mCherry::unc-54 3'UTR]*  *pmk-3(tm745);* *crgIs1006[Phsp-4::mCherry::unc-54 3'UTR]*  *lgg-1(bp500)II;xdIs13*  *daf-2(e1370);xdEx2318(Punc-25::mCherry::LGG-1)*  *daf-2(e1370);xdIs15;xdEx2318(Punc-25::mCherry::LGG-1)*  *xbp-1(zc12);daf-2(e1370);xdIs15;xdEx2318(Punc-25::mCherry::LGG-1)*  *pmk-3(tm745);daf-2(e1370);xdIs15;xdEx2318(Punc-25::mCherry::LGG-1)*  *ire-1(v33);daf-2(e1370);xdIs15;xdEx2318(Punc-25::mCherry::LGG-1)*  *ire-1(v33);xdEx2311 (Punc-25::hsp-4)*  *xbp-1(zc12);xdEx2312 (Punc-25::hsp-4)* |
